# Cationic liquid crystalline nanoparticles for the delivery of synthetic RNAi-based therapeutics

**DOI:** 10.18632/oncotarget.18421

**Published:** 2017-06-09

**Authors:** Emanuela Gentile, Taro Oba, Jing Lin, Ruping Shao, Feng Meng, Xiaobo Cao, Heather Y. Lin, Majidi Mourad, Apar Pataer, Veerabhadran Baladandayuthapani, Dong Cai, Jack A. Roth, Lin Ji

**Affiliations:** ^1^ Section of Thoracic Molecular Oncology, Department of Thoracic & Cardiovascular Surgery, The University of Texas MD Anderson Cancer Center, Houston, TX 77030, United States; ^2^ Department of Biostatistics, The University of Texas MD Anderson Cancer Center, Houston, TX 77030, United States; ^3^ Department of Physics, The University of Houston, Houston, TX 77004, United States

**Keywords:** delivery systems, cationic nanoparticles, RNAi, lung cancer, gene silencing

## Abstract

RNA interference (RNAi)-based therapeutics have been used to silence the expression of targeted pathological genes. Small interfering RNA (siRNAs) and microRNA (miRNAs) inhibitor have performed this function. However, short half-life, poor cellular uptake, and nonspecific distribution of small RNAs call for the development of novel delivery systems to facilitate the use of RNAi. We developed a novel cationic liquid crystalline nanoparticle (CLCN) to efficiently deliver synthetic siRNAs and miRNAs. CLCNs were prepared by using high-speed homogenization and assembled with synthetic siRNA or miRNA molecules in nuclease-free water to create CLCN/siRNA or miRNA complexes. The homogeneous and stable CLCNs and CLCN-siRNA complexes were about 100 nm in diameter, with positively charged surfaces. CLCNs are nontoxic and are taken up by human cells though endocytosis. Significant inhibition of gene expression was detected in transiently transfected lung cancer H1299 cells treated with CLCNs/anti-GFP complexes 24 hours after transfection. Biodistribution analysis showed that the CLCNs and CLCNs-RNAi complexes were successfully delivered to various organs and into the subcutaneous human lung cancer H1299 tumor xenografts in mice 24 hours after systemic administration. These results suggest that CLCNs are a unique and advanced delivery system capable of protecting RNAi from degradation and of efficiently delivering RNAi *in vitro* and *in vivo*.

## INTRODUCTION

RNA interference (RNAi) is a potential new class of drugs that can selectively silence disease-causing genes, including those causing genetic disorders, viral infections, autoimmune diseases, and cancer [1–4]. Two types of small RNA molecules are central to RNA interference: small interfering RNA (siRNAs) and microRNA (miRNAs) inhibitors and mimics [5–7]. Current efforts to introduce RNAi usage in the clinic involve the development of safe and effective systemic delivery systems that are stable in circulating blood and induce efficient cellular uptake [8, 9]. Based on the natural process of cell infection and the transfer of genetic materials into host, viruses have been evaluated as possible gene carriers, but toxicity, immunogenicity, and the inadequate size of the inserted genetic materials, impair their efficacy *in vivo* [10, 11]. To overcome these challenges, nonviral vectors such as lipid-based delivery systems, cationic liposomes, lipid nanoparticles, and a variety of cationic and biodegradable polymers [12, 13] have been used to mask the negative charges of the siRNA or miRNA backbone and facilitate cellular uptake, partially mediating the efficient delivery of siRNA *in vitro* and *in vivo* [14, 15]. Cationic liquid crystalline nanoparticles (CLCNs) are presented in this study as an advanced delivery system, for delivering siRNA or miRNA mimics *in vitro* and *in vivo* to either induce gene silencing or increase gene expression upon transfection [16]. CLCNs display several advantages including small size, decreased toxicity, longer half-life in circulation, and prolonged delivery over time. CLCNs also minimize nonspecific opsonization, phagocytosis, and immune activation and promote interaction with the cellular surface [17]. Furthermore, the fabrication method provides an efficient, cost-effective process for producing RNAi delivery systems.

CLCNs were produced by high-speed homogenization and successfully conjugated with siRNA or miRNA based on electrostatic interaction with the cationic lipid, 1, 2-dioleoyl-3-trimethylammonium-propane (chloride salt) (DOTAP). Results showed that the lowest concentration of DOTAP can induce efficient binding between the carrier and the RNAi and reduce toxicity *in vitro* and *in vivo*. CLCNs developed in this study offer an alternative approach for delivering siRNA or miRNA with the advantages of being prepared from physiologically well-tolerated materials and of having an efficient delivery system to silence or activate gene expression *in vitro* and *in vivo*.

## RESULTS

### Generation and characterization of CLCNs

**Figure 1:**
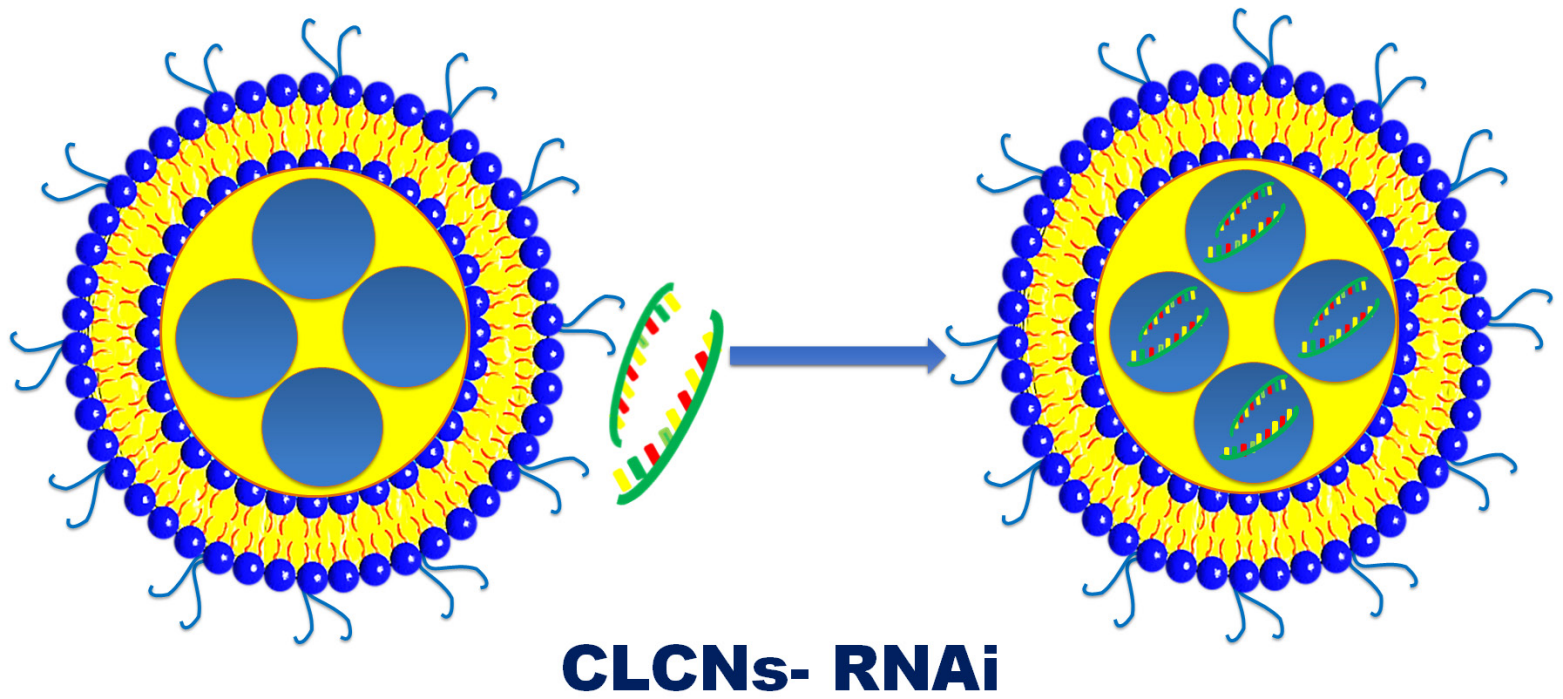
Schematic representation of CLCNs- RNAi binding CLCNs were prepared by using high-speed homogenization and assembled with synthetic siRNA or miRNA molecules in nuclease-free water to create CLCN/siRNA or miRNA complexes.

Cationic liquid crystalline nanoparticles (CLCNs) (Figure 1), were prepared by mixing together a lipophilic phase with a hydrophilic phase with use of high-speed homogenization. The lipophilic phase was made of a cationic phospholipid such as 2 -dioleoyl-3-trimethylammonium-propane (chloride salt) (DOTAP) to promote retention of the negatively charged RNAi in the core through electrostatic interaction and to control release of the RNAi and glyceryl monooleate such as 1-(cis-9-octadecenoyl)-rac-glycerol (GMO) to facilitate efficient interaction and fusion with the cell membrane. The hydrophilic phase was prepared dissolving in UltraPure DNase/RNase-Free Distilled Water a nonionic surfactant such as Pluronic F-127 to increase sustained release and to reduce degradation or dissociation of the CLCNs. After the homogenization and the purification, CLCNs were conjugated with nucleic acids, such as siRNA or miRNA therapeutics dissolved in UltraPure DNase/RNase-Free Distilled Water. A 1: 1 volume *ratio* between a calculated concentration of CLCNs and RNAi was used for both *in vitro* and *in vivo* experiments.

### CLCN1 and CLCN2

One of the major problem in the use of cationic nanoparticles is the high toxicity *in vitro* and *in vivo* due to the high concentration of cationic lipid such as DOTAP used to have positive charged nanoparticles, able to bind with the negative charged RNAi. To moderate DOTAP toxicity [18], two CLCN formulations, CLCN1 and CLCN2, were prepared based on the same reagents but with different *molar ratios* between GMO and DOTAP. Basically, the formulation CLCN1 had a higher DOTAP percentage of ~18% (wt) than the formulation CLCN2 where the DOTAP percentage was of ~7% (wt). For both formulations, the Pluronic concentration was 0.5% (w/v). CLCN1 and CLCN2 were tested in the same *in vivo* and *in vitro* experiments to check the most favorable combination to enable efficient delivery and low toxicity.

### Transmission electron microscopy (TEM)analysis

**Figure 2:**
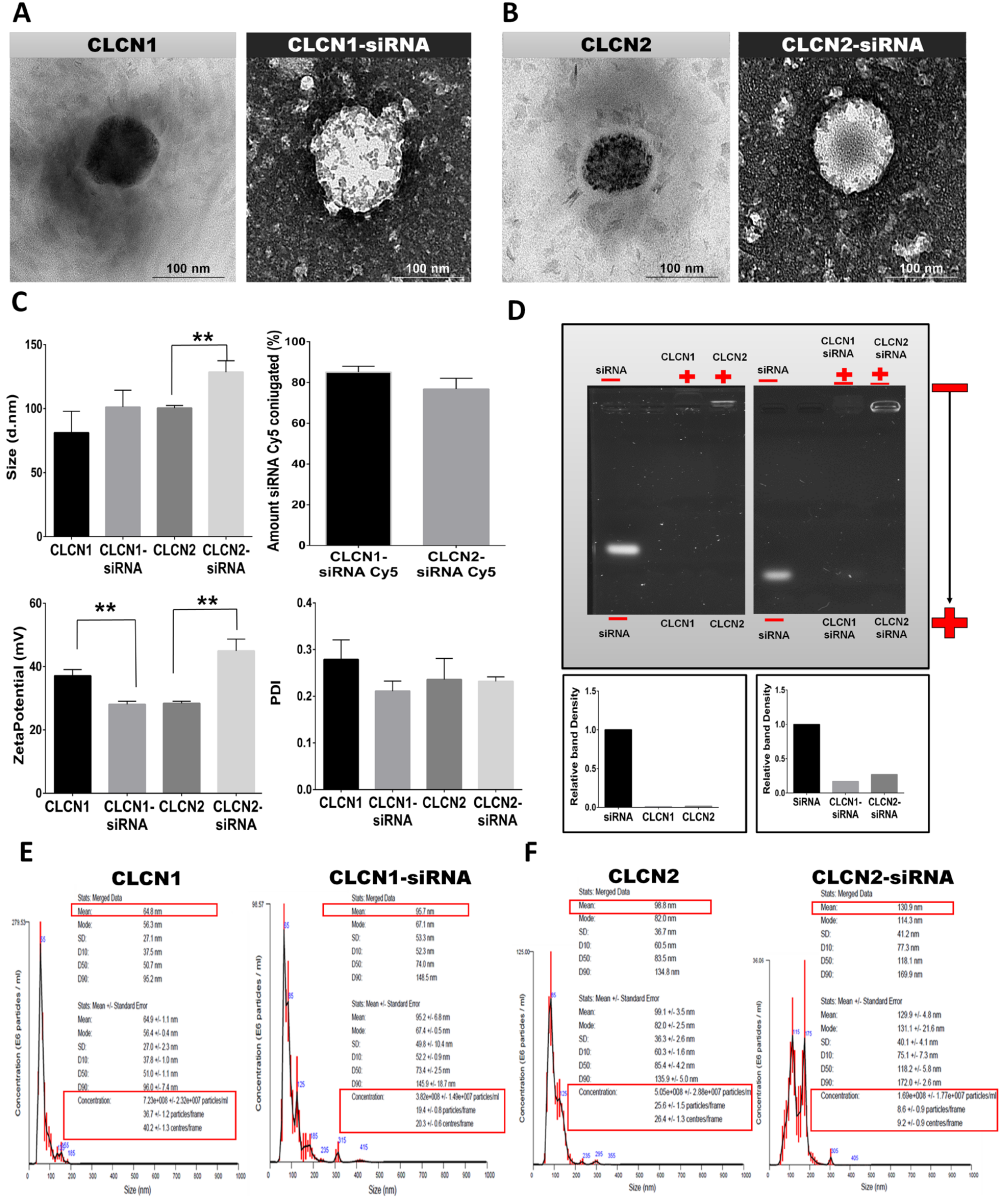
Physicochemical characterization of CLCNs and CLCNs/siRNA complexes **(A)** Transmission electron microscope images of CLCN1 and CLCN1 after conjugation with siRNA. **(B)** Transmission electron microscope images of CLCN2 and CLCN2 after conjugation with siRNA. **(C)** Physicochemical characterization (size of CLCNs and CLCNs/siRNA, zeta potential of CLCNs and CLCNs/siRNA complex and polydispersity index [PDI] by dynamic light scattering. Amount of siRNA conjugated by fluorescence analysis (%). Size: (**) p value 0.0063; zeta potential: (**) p value 0.0022 and (**) p value 0.0016 (unpaired two-tailed Student t test). **(D)** Gel retardation assay to evaluate the nanoparticle retardation inside the gel and the siRNA condensation inside the CLCNs and relative density of the bands (fold change value). **(E)** CLCN1 and CLCN1-siRNA complex size distribution per milliliters of solution using a NanoSight instrument for NTA (Table 1). **(F)** CLCN2 and CLCN2-siRNA complex size distribution per milliliters of solution using a NanoSight instrument for NTA (Table 1).

Morphological investigation was performed with a Transmission Electron Microscopy (TEM) operating at 80 kV (Figure 2A, 2B). TEM is a vital characterization tool for directly imaging nanomaterials to obtain quantitative measures of particle and/or grain size, size distribution, and morphology. A few microliters of the CLCN formulations were scanned using magnifications of 200000x and resolution of 100 nm and the images were recorded. The Figure 2A shows the formulation CLCN1 alone and conjugated with the siRNA. The Figure 2B shows the formulation CLCN2 alone and conjugated with the siRNA. CLCN1 and CLCN2 alone and conjugated with siRNA appear monodisperse systems with no sign of agglomeration but in both CLCNs conjugated with the siRNA homogenous and round spheres and core-shell structures are distinguishable. On the basis of these micrographs the following hypothesis may be drawn: the lighter color in the middle of the CLCN1-siRNA and CLCN2-siRNA should indicate the presence of the water channels contained the siRNA.

**Table 1:** Mean size and size distribution of CLCNs from NTA

Formulation	Mean(nm)	SD (nm)	Particle *conc*. (E8/ml)
CLCN1	64.9±1.1	27.0± 2.3	7.23
CLCN2	99.1±3.5	36.3±2.6	5.05
CLCN1-siRNA	95.2±6.8	49.8±10.4	3.82
CLCN2-siRNA	129.9±4.8	40.1±4.1	1.69

Mean and standard deviation (SD) calculated by NTA software; concentration (*CONC*.) in particles E8/ml as measured by NTA. Numbers represent average values ± standard deviation (n= 3 measurements).

### Physicochemical characterization of CLCNs

The quantitative physicochemical characterization of CLCNs was conducted with use of dynamic light scattering (DLS) to determine the size and homogeneity of the CLCNs (Figure 2C), and a Zetasizer Nano Z to measure the zeta potential (Charge) of the nanoparticles surface (Figure 2C). The physicochemical analysis revealed that CLCNs alone have a diameter ranging from 60 to 100 nm, with CLCN1's at about 70 nm and CLCN2's at about 90 nm. CLCNs conjugation with siRNA did not affect overall particle size, but CLCN2 conjugated with siRNA showed larger size (at about 100 nm) than CLCN1 conjugated with same siRNA (at about 90 nm) (Figure 2C size). CLCNs were homogeneous and stable nanoparticles, as demonstrated by a very low polydispersity index (PDI) ranging from 0.10 to 0.20 (Figure 2C PDI). In particular, both formulations displayed a lower PDI when conjugated with siRNA, confirming homogenous and monodisperse shape and structure shown in the TEM analysis (Figure 2A, 2B). The positive charge on the CLCN surface was between +25 and +35 mV. For CLCN1 and CLCN2 alone, the surface charges were, respectively, ~+35 and ~+30 mV (Figure 2C zeta potential). When CLCN1 and CLCN2 were conjugated with siRNA, the surface charges were, respectively, ~+30 and ~+45 mV (Figure 2C zeta potential). If all of the particles in suspension have a large positive zeta potential, they will not tend to aggregate or to flocculate. Particles with zeta potentials that are more positive than +30 mV are normally considered stable. The surface charge did not change from positive to negative after siRNA conjugation, suggesting complete internalization of the RNAi within the hydrophilic core and a stable nanoparticle suspension (Figure 2C zeta potential). The amount of RNAi conjugated to the CLCNs was measured using a red fluorescent siRNA (Cy5). Briefly, after the conjugation process the CLCNs-siRNA Cy5 were placed in 3K ultra centrifugal filter unit and centrifuged. The ultra-filtrate contained the free siRNA Cy5 was measured at a wavelength of excitation 650 nm and emission 670 nm. A standard curve was used to determinate the amount of siRNA form the fluorescence intensity. The amount of siRNA Cy5 conjugated to the CLCNs was calculated subtracting the amount of siRNA Cy5 added during the preparation procedure to the amount of free siRNA Cy5 found after the centrifugation. The results was around 80% for both formulations. (Figure 2C amount siRNA Cy5 conjugated (%)).

### Evaluation of the retardation of RNAi by CLCNs

Gel retardation assays were performed to evaluate the nanoparticle retardation inside the gel and the siRNA condensation inside the CLCNs (Figure 2D). Electrophoresis in 1% Agarose gels were carried out at 100 V for 20 minutes. A calculated amount of free siRNA was used as standard control and the same concentration was conjugated to the CLCN formulations (CLCN1-siRNA and CLCN2-siRNA); CLCN formulations alone were also loaded in the gel (CLCN1 and CLCN2). After electrophoresis, the gels were analyzed with use of a gel imaging system. The relative density of the bands was calculated to quantify the nanoparticles retardation inside the gel and the siRNA condensation inside the CLCNs, using ImageJ software (1.46r, http://imagej.nih.gov/ij). Basically, the percentage of the Area for each peaks resulted from the Agarose gel analysis was calculated and the Percent value for each sample (CLCN1 and CLCN2; CLCN1-siRNA and CLCN2-siRNA) was divided by the Percent value for the standard (free siRNA) to obtain the relative band density (fold change value). The condensation of siRNA inside the CLCNs was around 80% for both formulations indicating that the binding between the carrier and the siRNA was strong enough to withstand dissociation during electrophoresis, whereas the siRNA not complexed into CLCNs was free to run on the bottom of the agarose gel (Figure 2D).

### Nanoparticle tracking analysis (NTA)

To visualize, measure, and count the nanoparticles a Nanoparticle tracking analysis (NTA) was performed (Figure 2E, 2F) (Table 1). In this analysis, each nanoparticle in solution is individually but simultaneously analyzed by direct observation and measurement of diffusion events, producing high-resolution results for particle size distribution and concentration [19]. The mean size values obtained with the NTA were in the same range as those obtained by DLS analysis. NTA showed a higher particle concentration when CLCNs were alone. Specifically, CLCN1's concentration was ~7.23e+008 particles/ml, and CLCN2's was ~5.05e+008 particles/ml (Figure 2E, 2F) (Table 1); however, CLCNs complexed with siRNA displayed a lower concentration: CLCN1-siRNA's was 3.82e + 008 particles/ml, and CLCN2-siRNA was 1.69e+008 particles/ml (Figure 2E, 2F) (Table 1).

### Cellular uptake and processing of CLCNs in H1299

**Figure 3:**
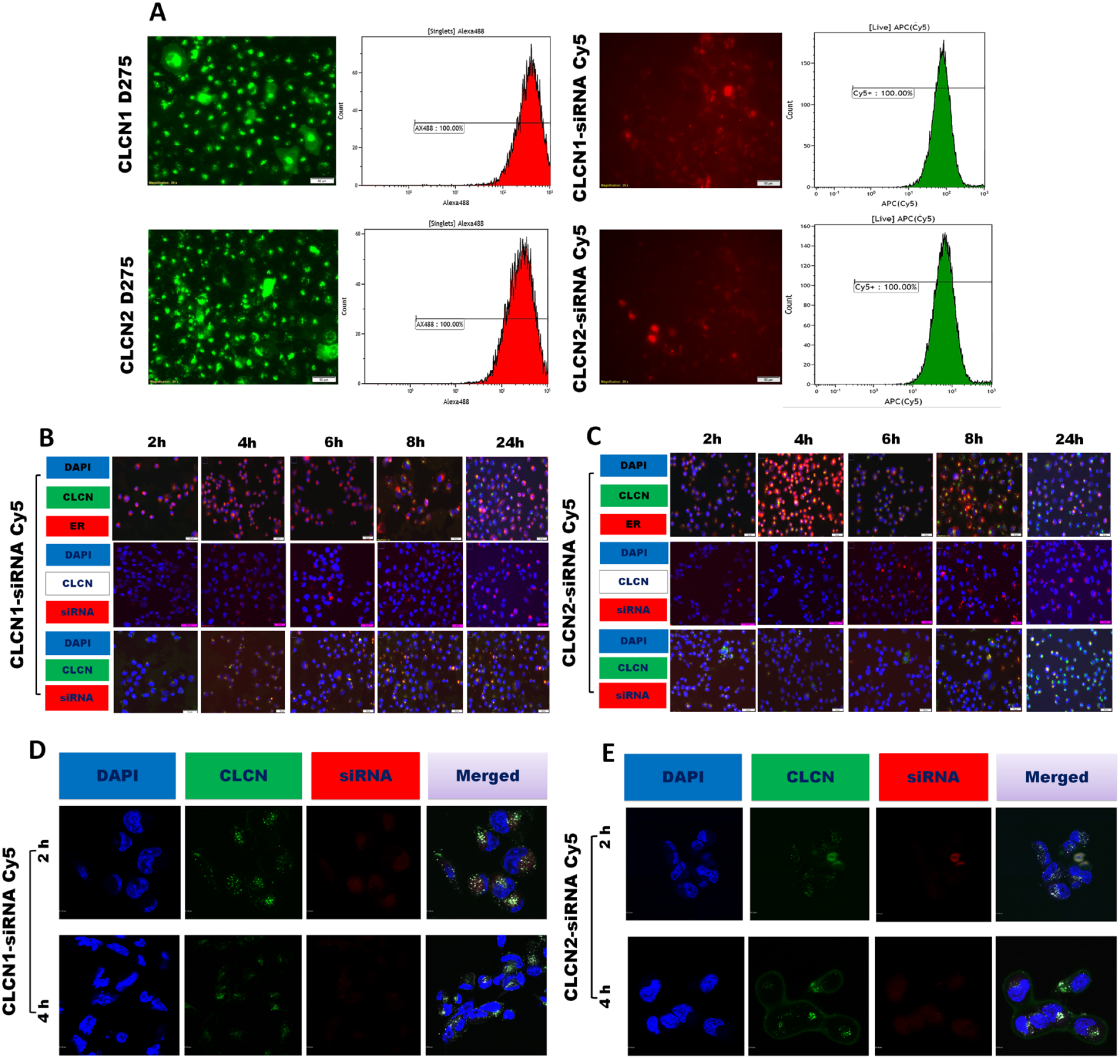
Cellular uptake by flow cytometry analysis and fluorescence microscopy image analysis **(A)** Fluorescence microscopy images, flow cytometry intensity analysis, and fluorescence intensity quantification after 24 hours of treatment with CLCNs D275 (green fluorescence) and CLCNs conjugated with siRNA Cy5 (red fluorescence) on H1299 cells. **(B)** Fluorescence microscopy images after 24 hours of treatment with CLCN1 D275 (green fluorescence) and CLCN1 conjugated with siRNA Cy5 (red fluorescence), ER, and CLCN1 D275/siRNA Cy5 complex and nucleus blue on H1299 cells. **(C)** Fluorescence microscopy images after 24 hours of treatment with CLCN2 D275 (green fluorescence) and CLCN2 conjugated with siRNA Cy5 (red fluorescence), ER, and CLCN2 D275/siRNA Cy5 complex and nucleus blue on H1299 cells. **(D)** Subcellular localization of CLCN1-siRNA by confocal fluorescence imaging analysis. The H1299 cells were treated with green fluorescence (D275) CLCN1, CLCN1/siRNA Cy5 complex (siRNA red fluorescence [Cy5]), and nucleus blue (DAPI). **(E)** Subcellular localization of CLCN2-siRNA by confocal fluorescence imaging analysis. The H1299 cells were treated with green fluorescence (D275) CLCN2, CLCN2/siRNA Cy5 complex (siRNA red fluorescence [Cy5]), and nucleus blue (DAPI).

The kinetics of internalization and intracellular trafficking of nanoparticle formulations were subsequently analyzed. As readout for monitoring the delivery of CLCNs, green fluorescent (D275) nanoparticles were prepared by using a fluorescent lipophilic tracer in the lipophilic phase and fluorescent and not fluorescent CLCNs were complexed with red fluorescent (Cy5) siRNA. In the Figure 3A the quantification of the fluorescent signal intensity was quantified by flow cytometry analysis and fluorescence microscopy images were taken. 24 hours after treatment on H1299 cell lines, the green fluorescent CLCNs 1 and 2 were able to diffuse into cells and release red fluorescent siRNA in the cytoplasm (Figure 3A).

### Fluorescence microscopy analysis

To better analyze the kinetics of cellular uptake and processing of the CLCNs, fluorescence microscopy images were taken at 2, 4, 6, 8, and 24 hours after treatment (Figure 3B, 3C). Green fluorescent (D275) CLCNs were prepared and fluorescent and not fluorescent CLCNs were complexed with red fluorescent (Cy5) siRNA. Various markers were used to determine the localization of CLCNs for each time point (Figure 3B, 3C). In the first lines in Figure 3B, 3C, H1299 cells were treated with green fluorescent CLCNs D275 and at each time point after treatment stained for the nucleus (DAPI, blue) and endoplasmic reticulum (ER, ER-Tracker^™^ red). Both formulation were able to diffuse in the cytoplasm but the formulation CLCN2 (Figure 3C) escaped faster from the ER than the formulation CLCN1 did (Figure 3B). In the second lines in Figure 3B, 3C, to analyze the kinetics of siRNA release in the cytoplasm, cells were treated with no fluorescent CLCNs complexed with fluorescent siRNA-Cy5 and counterstained for the nucleus with DAPI. The fluorescent signal of siRNA-Cy5 was already visible after 2 hours and increased over time in both formulations (Figure 3B, 3C). Finally in the third lines, cells were treated with green fluorescent CLCNs complexed with red fluorescent siRNA and counterstained for the nucleus with DAPI. Data showed green fluorescent CLCNs and the red fluorescent siRNA were dispersed in the cytoplasm after 2-4 hours (Figure 3B, 3C) as the previous fluorescence microscope images displayed.

### Confocal microscopy images

Confocal microcopy images were performed to confirm the mechanism of uptake and internalization of the CLCNs and the release of siRNA in the cytoplasm. CLCNs were labeled with green fluorescent lipophilic tracers (D275) and conjugated with red fluorescent siRNA (Cy5). H1299 tumors cells were treated for 2 and 4 hours with CLCNs D275- siRNA Cy5. After 2 and 4 hours DAPI blue was used to label the nucleus and confocal images were taken. In the Figure 3D, 3E single color channels (blue, green and red channels) and all channels overlapped together in one single image (merged) are shown. The confocal images confirmed the results previously reported at the same time points with the fluorescence microscopy, the green CLCNsD275 were dispersed in the cytoplasm and able to release the red siRNA Cy5 2 hours after treatment on H1299 cells (Figure 3D, 3E).

These results suggest that CLCNs are able to deliver siRNA after interaction with the cellular membrane and release it in the cytoplasm as early as 2 hours after treatment.

### Intracellular trafficking of CLCNs in H1299 cells by TEM

**Figure 4:**
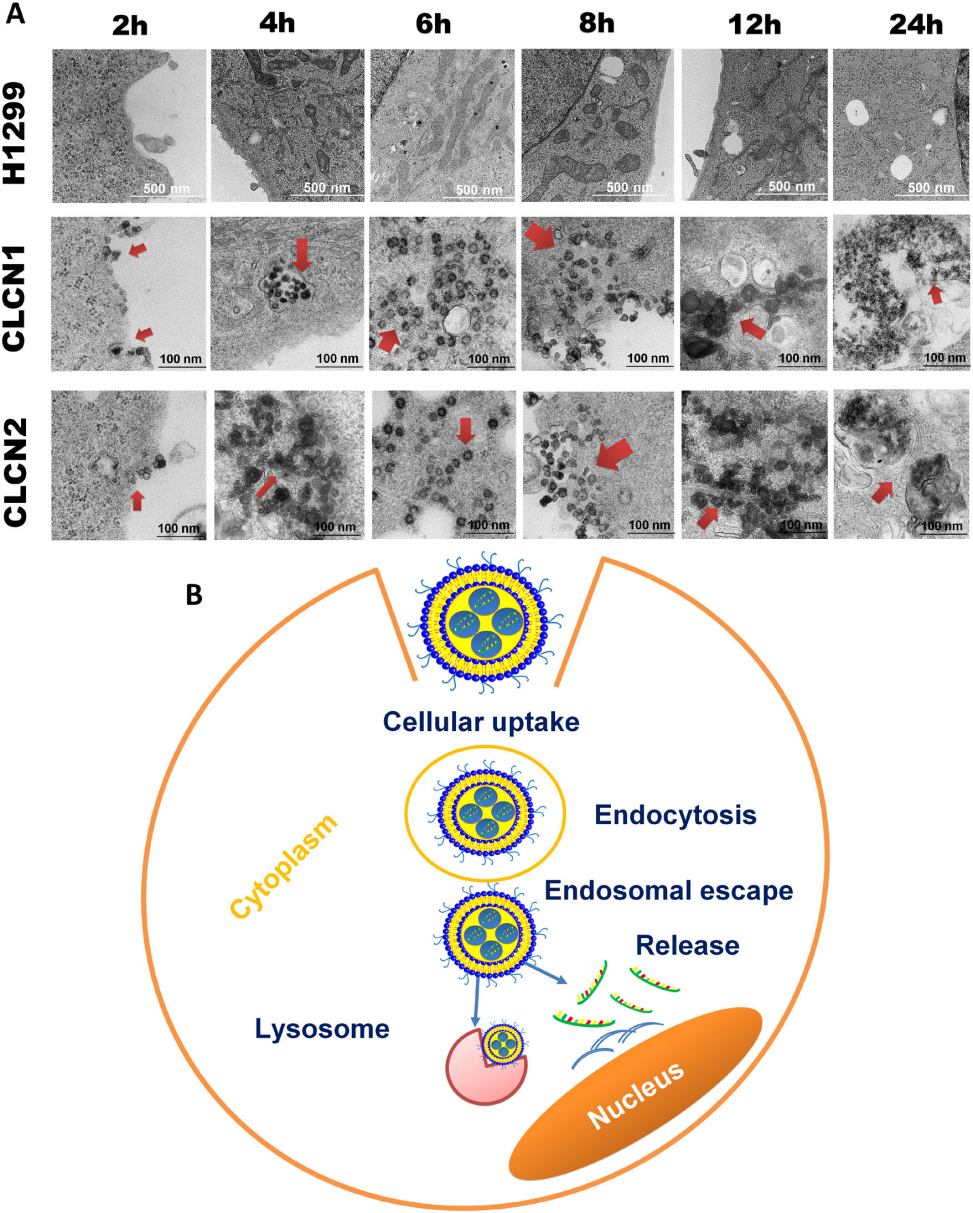
Intracellular trafficking of CLCNs in H1299 cells by TEM **(A)** Cellular uptake and processes of CLCNs in H1299 cells by TEM analysis after 2, 4, 6, 8, 12, and 24 hours of treatment. **(B)** Schematic representation of cellular uptake and internalization of CLCNs on the cell surface.

Further intracellular trafficking analysis conducted by TEM (Figure 4A) showed that CLCNs are taken up through endocytosis upon binding with the cell membrane and travel from early endosomes to the lysosome after 24 hours. The results also highlighted that CLCNs adhering to the plasma membrane were subsequently internalized by a vesicle-mediated endocytosis process. Nanoparticles located outside the endosomes were also observed at 6 hours and 8 hours. This further emphasizes the ability of CLCNs to escape endolysosomal entrapment shortly after intracellular uptake (Figure 4A). Thus, CLCNs are able to get inside the cells, escape from the ER, and release siRNA in the cytoplasm (Figure 4B).

### CLCNs RNAi mediated gene-silencing and gene-expression evaluation *in vitro*

**Figure 5:**
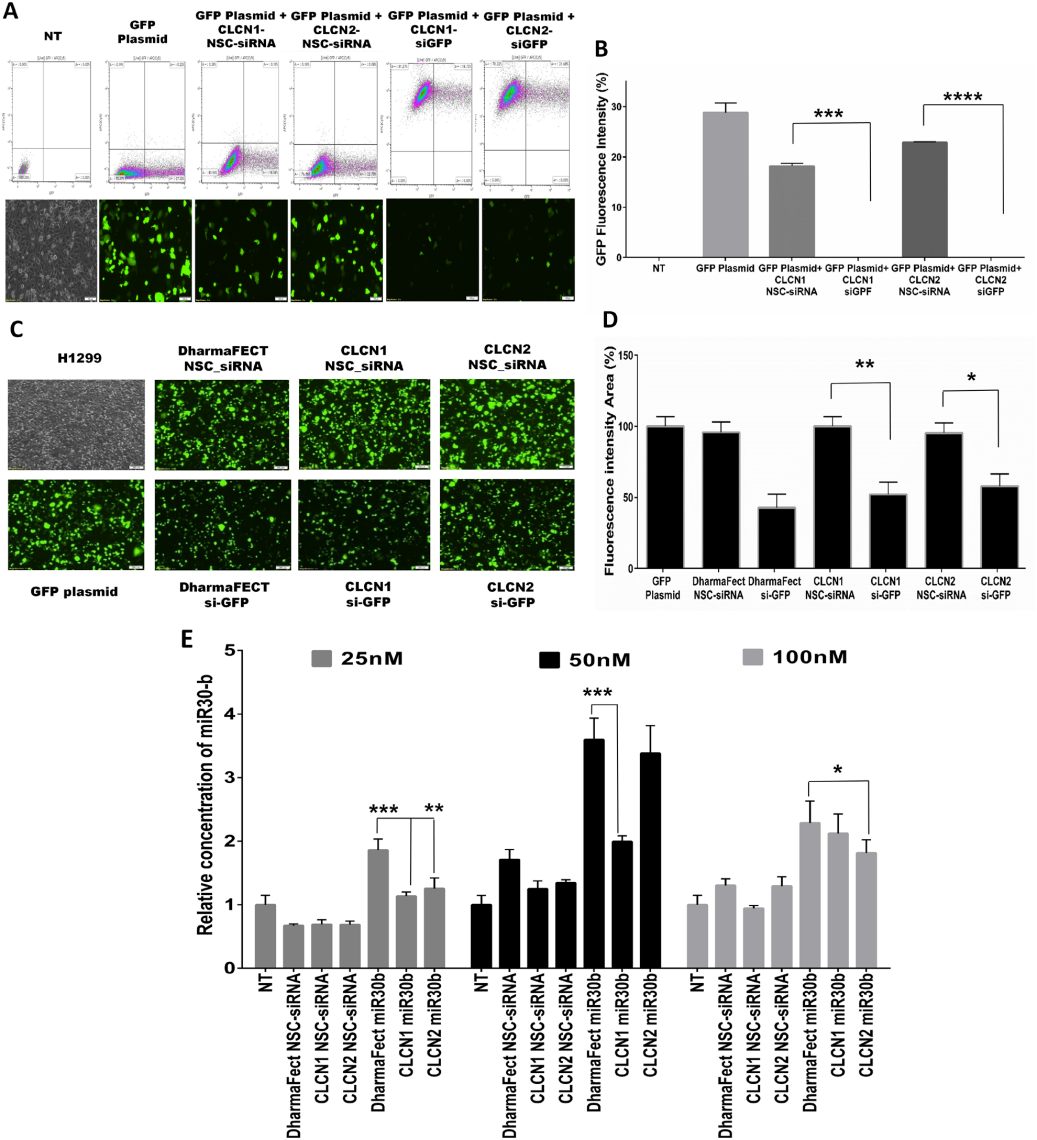
CLCNs RNAi mediated gene-silencing and gene-expression evaluation **(A)** Silencing of GFP expression in H1299 cells cotransfected with GFP plasmid vectors and CLCNs-siGFP nanoparticles for 24 hours. Flow cytometry analysis and fluorescence microscope images showed a dramatic reduction in GFP fluorescence when H1299 cells were transfected with anti-GFP siRNA complexed to the CLCNs formulations. (***) p value 0.0005; (****) p value < 0.0001 (unpaired two-tailed Student t test). **(B)** Graphical representation of the GFP fluorescence intensity percentage detected with flow cytometry analysis. (**) p value 0.0019; (*) p value 0.0203 (unpaired two-tailed Student t test). **(C)** Silencing of GFP expression in H1299 cells cotransfected with GFP plasmid vectors and CLCN-siGFP nanoparticles for 24 hours by fluorescence microscopy images. DharmaFect was used to compare CLCN transfection efficiency. **(D)** Calculation of GFP fluorescence intensity in the area of the images by ImageJ software (1.46r, http://imagej.nih.gov/ij). The fluorescence intensity percentage in the cells treated with only GFP plasmid was used as a control to compare the fluorescence intensity of the samples. **(E)** Quantification of cellular uptake of CLCNs-miR30b complexes by qRT-PCR at various miR30b concentration (25, 50, and 100 nM) after 24 hours of transfection. 25 nM, CLCN1-miR30b vs DharmaFect-miR30b p value 0.0003 (***) and CLCN2miR30b vs DharmaFect-miR30b p value 0.0053 (**). 50 nM, CLCN1miR30b vs DharmaFect-miR30b p value 0.0004 (***) and CLCN2miR30b vs DharmaFect miR30b p value 0.0225 (*).

Gene-silencing and gene expression evaluation experiments were performed to determine whether CLCNs are able to deliver siRNA to cells to induce silencing of a reporter gene (Green fluorescent protein, GFP) or enhancing the expression of an endogenous microRNA (miR-30b) (Figure 5). To this purpose, in the gene–silencing experiments, H1299 cells were cotransfected for 24 hours with a GFP plasmid conjugated with Lipofectamine 2000 and CLCNs/anti-GFP siRNA (si-GFP) complexes or CLCNs/NSC-siRNA as negative control siRNA, and flow cytometry and fluorescence microscopy analyses were conducted. When the H1299 were transfected with the GFP plasmid only or cotransfected with the GFP Plasmid and the CLCNs/NSC-siRNA, a high GFP transfection efficiency was achieved, whereas in the cells cotransfected with the GPF plasmid and CLCNs/anti-GFP siRNA (si-GFP) complexes, the GFP fluorescence intensity measured by flow cytometry analysis was dramatically reduced (***) p value 0.0005 and (****) p value < 0.0001 (Figure 5A, 5B). The experiment was repeated two times and the silencing efficiency of CLCNs was compared by fluorescence microscopy with that of the commercial transfection reagent DharmaFect (Dharmacon) (Figure 5C, 5D). Both CLCN formulations were able to deliver the anti-GFP siRNA and inhibit the GFP transfection as the DharmaFect did.

Gene expression evaluation *in vitro* was conducted on H1299 cells transfected with CLCNs conjugated with miR30b and the transfection efficiency was compared with that of the commercial transfection regent DharmaFect (Dharmacon) binding miR30b as well. Basically, H1299 were treated for 24 hours with various concentrations of miR30b, 25, 50 and 100 nM, conjugated with CLCNs or DharmaFect. As a negative control a scramble siRNA was used at the same concentrations and conjugated with CLCNs or DharmaFect. After 24 hours the cells were collected and the miR30b expression was evaluated by qRT-PCR assay. The results showed that CLCNs were able to transfect the cells with miR30b as well as DharmaFect did and the miR30-b expression *in vitro* was increased by using CLCNs or DharmaFect. Statistical analysis was performed to compare the transfection efficiency of CLCNs-miR30b vs DharmaFect-miR30b. Significantly different p-values were found at 25 nM concentration were the DharmaFect worked better than CLCNs above all the formulation CLCN1-miR30b vs DharmaFect-miR30b = 0.0003 (***) showed a lower transfection efficiency results. However CLCNs showed equivalent transfection efficiency with DharmaFect at higher concentration like 50 and 100 nM. All of these results suggested that CLCNs are able to efficiently transfect the cells *in vitro* and increase the miR30b expression similar to that seen with DharmaFect.

### Biodistribution of CLCNs by systemic administration and effect on gene expression in NSCLC tumor-bearing mice

**Figure 6:**
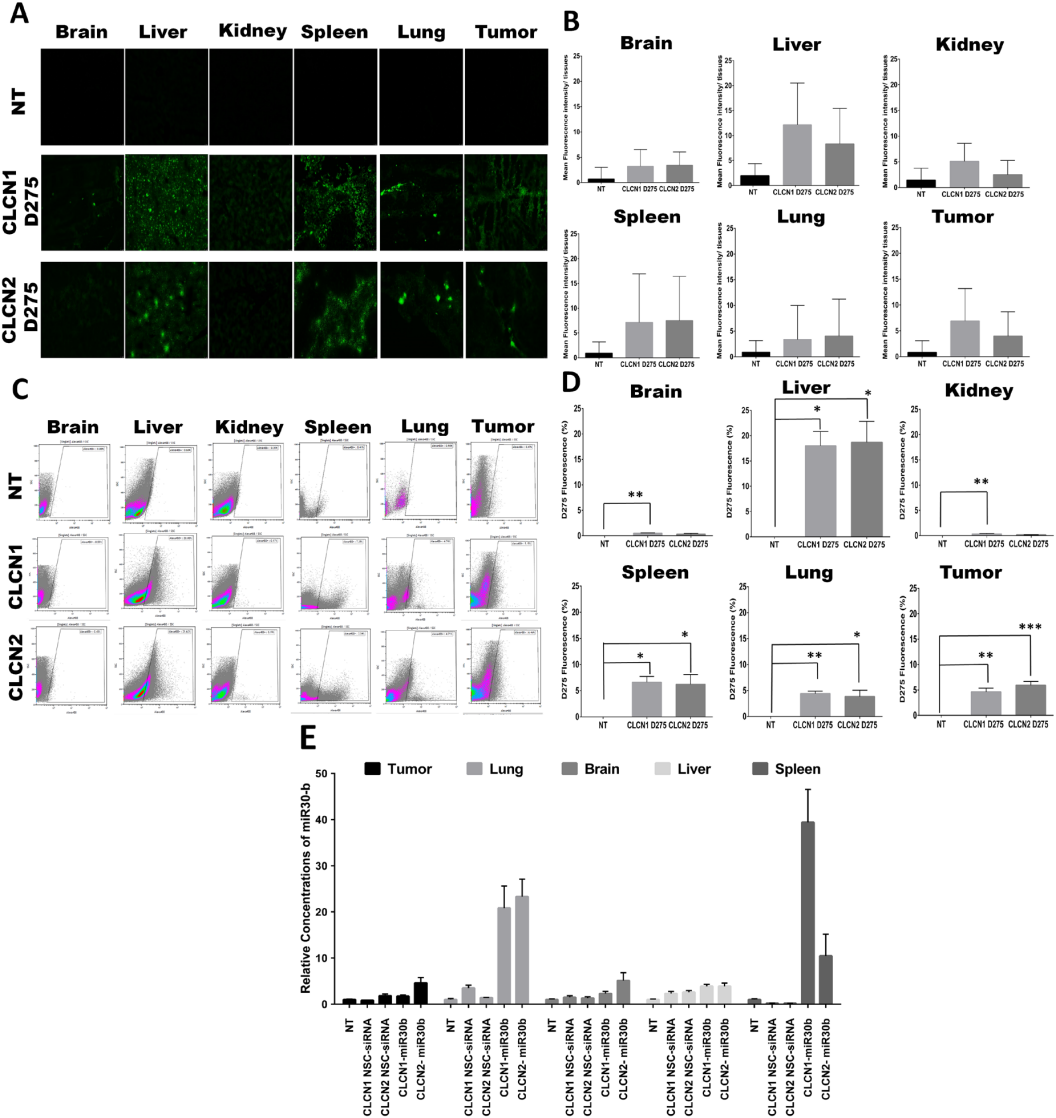
Biodistribution of CLCNs by systemic administration and effect on gene expression in NSCLC tumor-bearing mice **(A)** Images of fluorescence-labeled CLCNs in organs and tumors from mice 24 hours after tail vein injection. The tissue sections were collected after 24 hours of treatment with CLCNs D275 (10 mg/kg). The control group was not treated with CLCNs. **(B)** Quantification of fluorescence intensity by fluorescence microscopy images analysis by ImageJ software (1.46r, http://imagej.nih.gov/ij). **(C)** The fluorescence intensity of D275 encapsulated in the CLCNs was measured in various organs and tumors with use of flow cytometry analysis at a wavelength 460 nm excitation and 580 nm emission. **(D)** Representation of flow cytometry analysis fluorescence intensity percentage for each single tissue. Brain (**) p value 0.0078; liver (*) p value 0.0121 and (*) p value 0.0234; kidney (**) p value 0.0031; spleen (*) p value 0.0133 and (*) p value 0.0426; lung (**) p value 0.0063 and (*) p value 0.0477; tumor (**) p value 0.0013 and (***) p value 0.0007 (unpaired two-tailed Student t test). **(E)** Quantification with qPCR of miR30b expression in different mouse tissues. The tissues sections were collected 24 hours after treatment with CLCN D275/miR30 b complexes (1.5 mg/kg).

*In vivo* experiments were performed to evaluate CLCNs biodistribution and RNAi delivery. To study CLCNs biodistribution, nu/nu mice with H1299 subcutaneous tumors were injected intravenously with fluorescent CLCN1 D275 and CLCN2 D275 (10 mg/kg), and after 24 hours, the fluorescence intensity of the CLCNs was evaluated in tumors and major organs including liver, spleen, brain, lung, and kidney by fluorescence microscopy and flow cytometry analysis (Figure 6). Both fluorescence microscopy (Figure 6A, 6B) and flow cytometry analysis (Figure 6C, 6D) showed a higher signal in the liver, spleen, tumor, and lung for both CLCN formulations. In another experiment the same mice model was used to evaluate the effect on gene expression. CLCNs-miR30b complexes and CLCNs/negative siRNA control complexes were injected at a dose of 1.5 mg/kg via tail vein. Total RNA was extracted from tumors and major organs 24 hours later. The Quantitative real-time PCR showed a high concentration of miR30b in spleen and lung, liver and tumor (Figure 6E). These results suggest that CLCNs are able to reach the major organs like lung, liver spleen and the subcutaneous tumor and also if conjugated with a microRNA to deliver it to the major organs and increase the expression.

### Analysis of tumor growth rate after CLCN2-miR150 inhibitor administration

In this *in vivo* experiment a miR150 inhibitor was delivered intravenously using CLCN2 to treat H1299 human lung cancer xenografts. Tumor size was monitored for 3 weeks and statistical analysis of the tumor growth rate was performed using generalized linear mixed models. The tumor growth rate of the CLCN2-miR150 group was significantly lower than that of the control group (1.9% vs 18.0%, p<0.05, Table 2). These studies indicate that CLCN2 were able to efficiently deliver miR150 inhibitor and mediate suppression of tumor growth.

**Table 2:** Estimate of tumor growth rates by treatment groups

	Slope estimateon log2(tumor size)	Estimated tumor growth rateon raw scale
CLCN2-miR150	0.02667	1.9%
Control	0.2393	18.0%

Statistical analysis between the control group and the group treated with CLCN2-miR150 (n=5 mice for each group). P<0.05.

### CLCNs toxicity *in vitro* and evaluation of damages in organs function after CLCNs *in vivo* treatment

**Figure 7:**
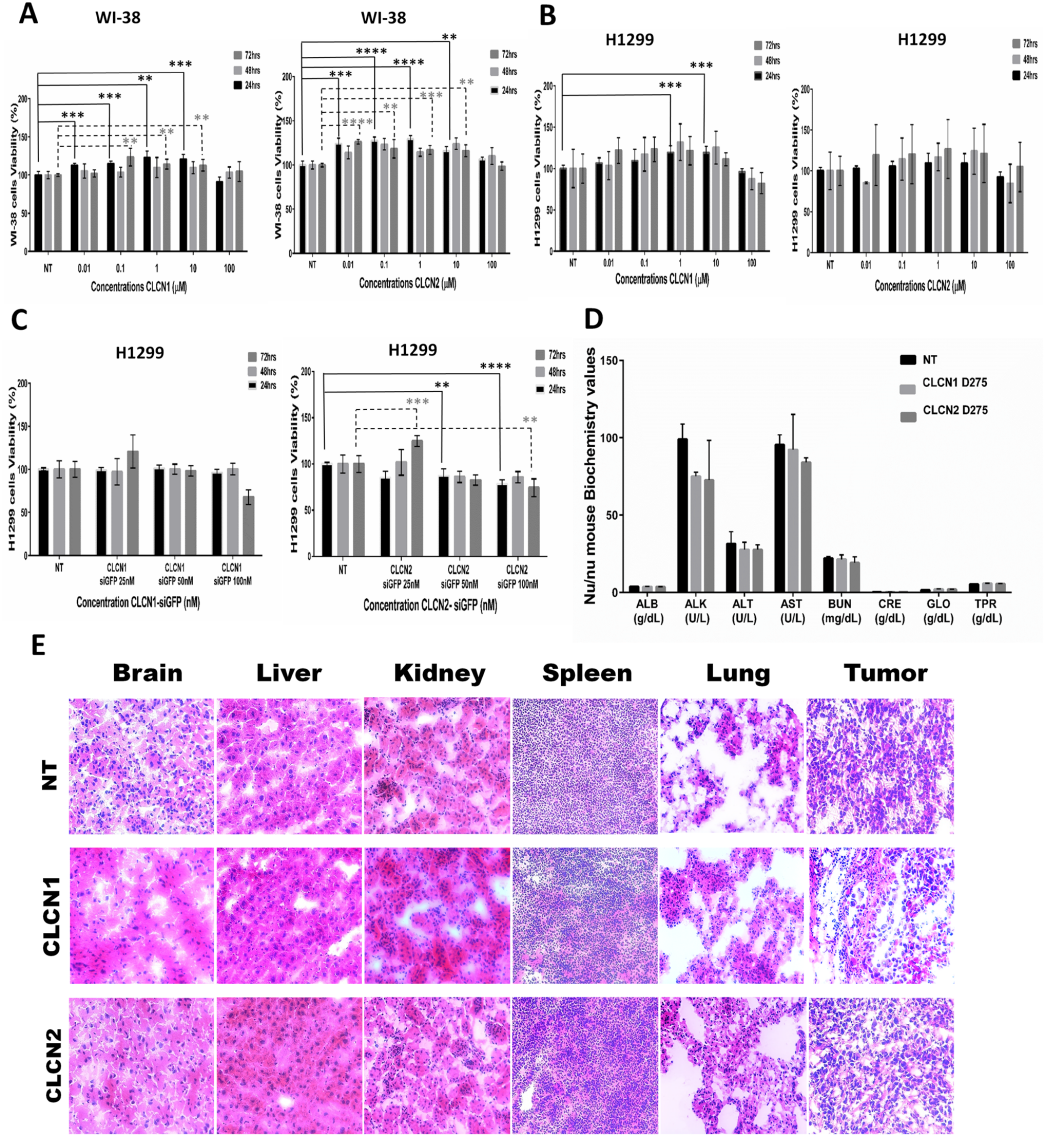
CLCNs toxicity *in vitro* and evaluation of damages in organs function after CLCNs *in vivo* treatment **(A)** Fibroblasts derived from lung tissue (WI-38) treated for 24, 48, and 72 hours with various CLCN concentrations ranging from 0.01 to 100 μM. Cytotoxicity was evaluated with the XTT assay. CLCN1 at 24 hours: (***) p value 0. 0013; (***) p value 0.0009; (**) p value 0.0013; (***) p value 0.0006. CLCN 1 at 72 hours: (**) p value 0.0026; (**) p value 0.0020; (**) p value 0.0081. CLCN2 at 24 hours: (***) p value 0.0003; (****) p value < 0.0001; (****) p value <0.0001; (**) p value 0.0019. CLCN2 at 72 hours: (****) p value < 0.0001; (**) p value 0.0060; (***) p value 0.0002; (**) p value 0.001 (unpaired two-tailed Student t test). **(B)** Non–small cell lung cancer (H1299) treated for 24, 48, and 72 hours with various CLCN concentrations ranging from 0.01 to 100 μM. CLCN1 at 24 hours: (***) p value 0.0004; (***) p value 0.0004 (unpaired two-tailed Student t test). **(C)** Non–small cell lung cancer (H1299) treated for 24, 48, and 72 hours with various CLCN-siRNA concentrations at 100 nM, 50 nM, and 100 μM. CLCN 2-siGFP at 24 hours: (***) p value 0006; (**) p value 0.0024; (****) p value <0.0001. CLCN2-siGFP at 72 hours: (***) p value 0.0004; (**) p value 0.0027 (unpaired two-tailed Student t test). **(D)** Biochemical values of mice blood 24 hours after CLCNs D275 systemic injection by tail vein. **(E)** Routine histopathology analysis, H&E staining of major organs after 24 hour of CLCNs injection at 10 mg/kg dose.

Cytotoxic effects of the nanoparticles were first tested *in vitro* in lung cancer (H1299) and normal fibroblast and bronchial epithelial cells (WI-38) (Figure 7). Varying concentrations of CLCNs, from 0.01 to 100 μM, were used to treat the cells, and cell viability and proliferation were evaluated after 24, 48, and 72 hours. The CLCNs were not toxic on normal cells WI-38 (Figure 7A) or H1299 tumor cells (Figure 7B). The cytotoxicity of CLCN-siRNA complexes was also evaluated on H1299 tumor cells (Figure 7C) at varying nM concentrations of siRNA (25, 50, and 100 nM). For *in vivo* studies, mice were treated with fluorescent CLCN1 D275 and CLCN2 D275 at a dose of 10 mg/kg by intravenous injection. After 24 hours, blood was collected from each mouse for a routine chemistry analysis to check liver or kidney function (Figure 7D); this analysis showed no liver or kidney damages, thus suggesting that CLCNs are not associated with any changes in hematological parameters or serum biochemical markers. A routine histopathology analysis (Figure 7E) was performed to check alterations in the major tissues after CLCNs treatment. Specifically, after 24 hours of CLCN1 and CLCN2 injection at 10 mg/Kg dose, all major organs and tissues were collected, and sections were stained for Hematoxylin and eosin stain. A no treatment group was used to compare the tissue anatomy and morphology with the treated groups. Pathologic review showed that there were no abnormalities or changes for all major organs and tissues after exposure of animals to 10 mg/kg dose of CLCN1 and CLCN2. The results obtained from the blood chemistry and the histopathology analysis indicated that the CLCNS are not associated with organ toxicity after 24 hours of treatment at the dose of 10 mg/Kg.

## DISCUSSION

RNA interference (RNAi), since its discovery in the 1990s, has rapidly become a useful tool for studying functional genomics and validating gene targets *in vitro* and *in vivo*, as well as for developing gene-specific medicines [20–23]. In order for synthetic RNAi-based therapeutics to be applied in a broad range of diseases, however, a number of challenges must be overcome [24]. Nanoparticle-based drug delivery systems can effectively deliver and release therapeutic payloads [25, 26], but to achieve efficient endocytosis and gene transfer, the complex must be small (less than 200 nm) and compact [27]. High toxicity, instability in the presence of serum and release from endosomes into the cytoplasm are the primary causes of poor and inefficient gene delivery and of limit applications *in vivo* [28, 29]. In this study novel cationic crystalline nanoparticles (CLCNs) for the delivery of RNAi are presented. Two CLCN formulations, CLCN1 and CLCN2, having a different *molar ratio* between the glyceryl monooleate (GMO) and the cationic phospholipids (DOTAP) in the lipophilic phase, were tested *in vitro* and *in vivo*. CLCNs were prepared based on the same reagents but CLCN2 formulation had a lower DOTAP percentage than that of CLCN1, in order to reduce the cationic phospholipids toxicity and achieve high transfection efficiency. The physicochemical characterization demonstrated that CLCNs are monodispersed delivery systems that are about 100 nm in diameter, with a lipid bilayer enclosing an aqueous core, surrounded by a more hydrophobic shell. CLCNs have a positively charged surface, and are able to bind with nucleic acids, such as siRNA or miRNA therapeutics and keep it inside the structure. As expected, we found that the percentage of RNAi conjugated to the CLCNs after preparation was about 80% and the gel retardation assay showed that the CLCNs were able to bind the negative charged RNAi enough to withstand dissociation during the electrophoresis. On explanation for this tight conjugation, is that the hydrophobic cationic material and the hydrophobic portion of the amphiphilic material provide a non-polar polymer matrix for loading, protecting, and promoting RNAi molecules retention and controlling the release.

A qualitative and quantitative cellular uptake and processing analysis demonstrated that the CLCNs are internalized by a vesicle-mediated endocytosis process and are able to deliver siRNA after interaction and fusion with the cellular membrane, escape from the endoplasmic reticulum (ER) and release it in the cytoplasm as early as 2 hours after treatment. *In vitro* experiments were performed to evaluate the efficacy of CLCNs to deliver the RNAi and achieve gene-silencing or gene expression. In gene-silencing experiments, lung cancer H1299 cells were transiently transfected with GFP plasmid and a significant inhibition of gene expression was detected when the cells were cotransfected with CLCNs/anti-GFP siRNA complexes. Untreated control group and non-specific CLCN-siRNA were used as negative control to validate the gene silencing efficiency of CLCNs/anti-GFP siRNA complexes. Both formulations were able to induce similar gene silencing efficacy when compared to the commercial transfection reagent DharmaFect. *In vitro* gene expression experiments were performed transfecting lung cancer cells, H1299, with CLCNs-miR30b complexes and the relative gene expression of miR30b was evaluated after 24 hour of transfection. miR30 is significantly down-regulated in several cancers, including breast cancer [30] and lung cancer [31] and it has been hypothesized that miR30 may play an important role in tumorigenesis and tumor development. However, the function of miR30 especially in NSCLC remains unclear [32]. The fold expression of miR30b was evaluated by RT-qPCR after 24 hours of treatment with CLCNs-miR30b complexes at various concentrations (25, 50 and 100 nM). The transfection efficiency of CLCNs was compared to that of DharmaFect conjugated miR30b at similar concentrations. Untreated control group and non-specific CLCN-siRNA were used as negative control to validate the gene silencing efficiency of CLCNs/miR30b complexes and Dhermafect-miR30b. CLCNs showed equivalent transfection efficiency to DharmaFect at the concentration of miR30b of 50 and 100 nM. Among non-viral vectors, cationic lipids such as Lipofectamine and DharmaFect have high transfection efficiencies *in vitro* but their high toxicity and instability in the presence of serum limit applications *in vivo*. Besides nanoparticles size, surface chemistry, and charge impact, shape, and structure can determine potential cytotoxicity both *in vitro* and *in vivo*. CLCNs were tested on lung cancer H1299 cells and normal fibroblast and epithelial cells (WI-38). Cytotoxic effects of CLCNs and CLCNs-siRNA complexes, were evaluated at various concentrations for 24, 48 and 72 hours. CLCNs are very safe and biocompatible, even when they were conjugated with RNAi. Biodistribution studies are necessary to provide preclinical safety evaluation and tacking of novel gene therapy technologies. *In vivo* experiments were designed to identify any changes in hematological parameters or serum biochemical markers and alterations in the tissues morphologies and function. The results obtained from routine chemistry analysis and routine histopathology analysis suggested that the CLCNs did not induce organs function alteration or tissues damages at the dose of 10 mg/Kg after intravenous injection. Biodistribution studies were performed to track fluorescent CLCNs (CLCNs D275) and to evaluate the expression of miR30b delivered by CLCNs, in the major organs and tissues after 24 hours of intravenous administration by tail vein. The fluorescent intensity evaluation of CLCNS D275 was estimated by fluorescence microscopy and flow cytometry analysis. The results from the two different method of fluorescence evaluation showed the same fluorescent intensity percentage in each organs. CLCNs were able to reach major organs such as liver, lung, spleen and the subcutaneous tumor after 24 hours of injection. In the microRNA biodistribution experiment no fluorescent CLCNs were conjugated with miR30b and the concentration of miR30b was quantified using RT-qPCR. Quantitative real-time polymerase chain reaction (qPCR) was the method of choice for the detection and quantitation of specific gene sequences or vectors in biodistribution studies. qPCR is a highly sensitive, analytical method for determining whether a nucleic acid (DNA or RNA) target is present in tissues. The expression of miR30b increased in the spleen, lung, liver, and in the tumor. CLCNs were able to reach the major organs after 24 hours of injection and release RNAi without damaging the tissues. All of these *in vitro* and *in vivo* studies demonstrated that the CLCNs are a promising strategy for delivering RNAi for therapeutic purpose [33, 34]. Among the two different formulations investigated in this study, CLCN2, having a lower percentage of DOTAP, exhibited better results in term of RNAi delivery and low toxicity *in vitro* and *in vivo*. The comparison between the two CLCN formulations showed that the relative amounts of cationic lipid and GMO can be used to control the conjugation of the cationic lipid with the RNAi, thus enhancing delivery efficacy and reducing toxicity. On the basis of these experimental findings, the formulation CLCN2 was conjugated to the tumor suppressor microRNA, miR150 inhibitor [35] and its therapeutic efficacy was evaluated *in vivo*. In previous studies, researchers identified that miR150 promotes the proliferation and migration of lung cancer cells through specifically targeting such as the 3’-UTR of p53, SRCIN1 and BAK1 [36, 37]. The inhibition of miR-150 expression effectively delayed cell proliferation and promoted apoptosis in the lung carcinoma cells [38]. Tree injections of CLCN2/ miR150 inhibitor were intravenous administrated to H1299 human lung cancer xenografts at the miR150 inhibitor dose of 1.5 mg/Kg. The tumor size was monitored for 3 weeks and a statistical analysis of the tumor growth rate was performed at the end of the experiment. CLCN2 were able to efficiently deliver the miR150 inhibitor which resulted in decrease of tumor growth rate as compared to the no treatment control group. CLCNs are unique and advanced delivery systems able to protecting and delivering the RNAi. Further studies are needed to determine the therapeutic efficacy of these novel nanoparticles.

## MATERIALS AND METHODS

### Materials

1-(cis-9-octadecenoyl)-rac-glycerol (monoolein, glyceryl monooleate, GMO content >99%), Pluronic F-127 was purchased from Sigma-Aldrich. 1, 2-dioleoyl-3-trimethylammonium-propane (chloride salt) (DOTAP) was purchased from Avanti Polar Lipids. These chemicals were used as received without further purification.

Cells from the H1299 (human non–small cell lung cancer) cell line were cultured in RPMI-1640 medium (HyClone) supplemented with 10% fetal bovine serum (FBS) (HyClone). The Wi-38 (normal bronchial epithelial cells) were cultured in Eagle's Minimum Essential Medium (EMEM) (Corning) supplemented with 10% FBS (Hyclone). Cells were grown at 37°C in a humidified atmosphere of 5% CO_2_ (v/v) in air. Cells were seeded at an initial density of 20%–25% confluence in 6-well plates or 60-mm or 100-mm culture dishes or chamber slides according to experimental procedures and grown for at least 24 hours before any treatment.

### Preparation of CLCNs

1-(cis-9-octadecenoyl)-rac-glycerol and 2-dioleoyl-3-trimethylammonium-propane (chloride salt) were solubilized in ethanol at different *molar ratios*. 25 mg of Pluronic F-127 was solubilized at 4°C in RNAsi-free water. The polymeric solution was added drop by drop to the lipophilic mixture under high-speed homogenization (IKA ULTRA-TURRAX T-25). The resulting solution was placed on a magnetic stirring plate for 24 hours for ethanol evaporation, after which the dispersion was stored at 4°C before further experimentation to enable equilibration of lipids, Pluronic, and water. Green fluorescent CLCNs were prepared by using a lipophilic tracer D275 (Invitrogen molecular probe) in the lipophilic phase at 0.01% (w/v). CLCNs at various *molar ratios* were conjugated with miRNA (Ambion, ThermoFisher scientific) or siRNA (Sigma-Aldrich) in sterile conditions. Briefly, for both *in vitro* and *in vivo* experiments, a 1: 1 volume ratio between a calculated concentration of CLCNs and RNAi was used. Red fluorescent siRNA–Cy5 (siRNA Fluorescent Universal Negative Control #1, Cyanine 5 Sigma-Aldrich) was conjugated to CLCNs for imaging experiments.

### CLCNs physicochemical characterization

CLCNs and the CLCNs complexed with siRNA or miRNA were analyzed by DLS measurements (ZetaSizer Nano ZS, Malvern Instruments) to retrieve information on size and polydispersion index (PDI), at a temperature of 25°C ± 0.1°C. About 20 μl of each nanoparticle suspension was diluted in water, housed in disposable polystyrene cuvettes of 1-cm optical path length, and backscattered by a 4 mW He–Ne laser (operating at a wavelength of 633 nm) at an angle of 173° (each sample was measured 5 to 10 times). The zeta potential was measured by using standard disposable Z potential flow cells after the particles were diluted in water (as neutral charged solution). All measurements were repeated three times at 25°C. The amount of RNAi conjugated to the CLCNs was measured after centrifugation in Amicon Ultra centrifugal Filters 3K (Millipore). The percentage of fluorescent siRNA (Cy5) was measured in the ultrafiltrate using a fluorescence-based microplate reader at a wavelength of excitation 650 nm and emission 670 nm. A standard curve was used to determinate the amount of siRNA from the fluorescence intensity.

### Evaluation of the retardation of miRNA by CLCNs

9 μl of CLCNs complexed with siRNA Cy5 was mixed with 1 μl of loading buffer (6x DNA Loading Thermo Scientific). The samples were loaded into a 1% (w/v) agarose gel containing 0.5 μg/ml ethidium bromide per well. Electrophoresis was carried out at 100 V for 20 minutes in Tris-acetate-EDTA (TAE) running buffer. After electrophoresis, the gel was analyzed with use of a gel imaging system. The relative density of the bands was calculated using ImageJ software (1.46r, http://imagej.nih.gov/ij).

### Nanoparticle tracking analysis (NTA)

Nanoparticle size and concentration were measured at the same time by using a “NanoSight NS300 Instrument (Malvern Instruments). A fluorescence mode provides differentiation of labeled or naturally fluorescing nanoparticles. The instrument uses a particle-by-particle system to produce high-resolution results for particle size, distribution, and concentration. The standard nanoparticle concentration in a diluted sample volume of ~1 ml was estimated to be about 10^6^-10^9^ particles/ml.

### Cellular uptake and processing of CLCNs in H1299

*In vitro* cellular uptake and processing of CLCNs were evaluated in tumor cell line H1299 by using quantitative and imaging methodologies. H1299 cells were seeded into a 6-well plate at 2 × 10^5^ cells/well and cultured overnight. Green fluorescent CLCNs (CLCNs D275 wavelength 460/580 nm) and CLCNs conjugated with red siRNA (CLCNs-siRNA Cy5 wavelength 650/670 nm) at a concentration of 100 nM were incubated with H1299 cells for 24 hours, and uptake was evaluated by fluorescence microscopy (with an Olympus IX81 microscope) or flow cytometry (Gallios Flow Cytometer). In fluorescence and confocal microscopy experiments, H1299 cells were seeded into a 4-chamber slide (Nunc^™^ LabTek^™^ II Chamber Slide^™^ System, ThermoFisher Scientific) at 2 × 10^3^. After 24 hours, cells were treated with CLCNs D275 or CLCNs-siRNA Cy5 for 2, 4, 6, 8, and 24 hours. For fluorescence microscopy images the nuclei were stained with DAPI blue (ThermoFisher Scientific) and the ER was stained with ER-Tracker^™^ (red fluorescence wavelength of 587/615) (ThermoFisher Scientific). For confocal microscope analyses, H1299 cells were fixed with PFA 4% and the nuclei were stained with DAPI blue. Confocal images were taken using a FV1000 Olympus Laser Confocal.

### Transmission electron microscope

Transmission electron microscope images (JEM-1010 Transmission Electron Microscope) were acquired to evaluate the morphology and the structure of CLCNs and the internalization of the CLCNs in the tumor cells at various time points (2, 4, 6, 8, 12, and 24 hours). A small drop of the CLCN formulations was deposited on the carbon coated grid, allowed to settle, blotted dry and then covered with a small drop of the negative stain. For the *in vitro* experiments H1299 cells were seeded in 35-mm culture dishes, treated after 24 hours with CLCN formulations, incubated with a fixative solution (2.5% glutaraldehyde in 0.1 M sodium cacodylate), and stored at 4°C. A small drop of the cell suspensions was deposited on the carbon coated grid and covered with a small drop of the negative stain. The images were acquired with use of a JEOL JEM-1010 transmission electron microscope equipped with digital cameras.

### GFP silencing assay

H1299 cells were seeded 2 × 10^5^ in 6-well tissue culture plates in triplicate and grown overnight at 37°C with 5% CO_2_. When 80% confluent, cells were transfected with 2.5 μg of GFP plasmid (pMAX-GFP) using Lipofectamine transfection reagent (Invitrogen). In a first experiment cells where cotrasfected with GPF Plasmid-Lipofectamine and CLCNs-anti-GFP Positive Control siRNA (siGFP) or CLCNs-negative control siRNA (Ambion® Silencer GFP) at a concentration of 100 nM. After 24 hours, cells were analyzed by fluorescence microscopy (Olympus IX81) and flow cytometry (Gallios Flow Cytometer). To this purpose, cells were washed with PBS, trypsinized, washed once with flow cytometry washing solution (PBS 3% FBS), and analyzed by flow cytometry. In another experiment anti-GFP Positive Control siRNA (siGFP) or negative control siRNA (Ambion® Silencer GFP) at a concentration of 100 nM were delivered by either CLCNs or DharmaFECT transfection reagents (Dharmacon) to compare silencing efficiency. After 24 hours, cells were analyzed by fluorescence microscopy (Olympus IX81) and the fluorescence intensity was calculated using, ImageJ software (1.46r, http://imagej.nih.gov/ij). GFP silencing was calculated as the percentage of GFP fluorescence intensity in samples treated with CLCNs-siRNA anti-GFP compared with control samples.

### Gene expression *in vitro*

Cells were seeded in 6-well plates at an initial density of 2 × 10^5^ cells/well. After 24 hours, the cells were treated with CLCNs conjugated with miR30b (Ambion) or DharmaFECT transfection reagents (Dharmacon) mixed with miR30b as well. Various concentrations of miR30b (25, 50, and 100 nM) were used and the same concentration were used for the NSC-siRNA (negative control) conjugated to CLCNs or to DharmaFect. After 24 hours total RNA was extracted from the samples by using TRIzol® RNA Isolation Reagents (Thermo Fisher Scientific). Reverse transcription was performed by using a High-Capacity cDNA Reverse Transcription Kit (Applied Biosystems) and miR30b RT primers. Quantitative real-time PCR (qPCR) was performed by using TaqMan® MicroRNA Assays (Life Technologies) on a CFX384 Real-Time System (Bio-Rad) and miR30b TM primers.

### *In vitro* cytotoxicity assay

Cells were seeded in 96-well plates at an initial density of 3 × 10^3^ cells/well. After 24 hours, cells were treated with various concentrations of CLCNs and CLCNs/siRNA Cy5, for 24, 48, and 72 hours at 37°C in a humidified, 5% CO_2_ atmosphere. The cytotoxicity at each time point was evaluated by using a standard 2, 3-bis (2-methoxy-4-nitro-5-sulfophenyl)-5-[(phenylamino) carbonyl]-2H-tetrazolium hydroxide (XTT) II assay (Sigma-Aldrich). Absorbance was determined on a plate reader at 492 nm. The percentage of cell viability was calculated according to the following equation:

$$cell\;viability\;\left(\%\right)=\frac{ABS\;T}{ABS\;C}x100$$

Where ABS T is the absorbance of treated cells and ABS C is the absorbance of control (no treated) cells.

### *In vivo* studies

Animal studies were approved by the Institutional Animal Care and Use Committee of The University of Texas MD Anderson Cancer Center and performed according to NIH guidelines. Female nude mice (nu/nu), aged 4-6 weeks, were purchased from the Charles River Company. Before any experiment were started, the mice were acclimatized for 5 days in the animal core facility.

### *In vivo* fluorescent CLCNs D275 biodistribution

H1299 cells were injected into nu/nu female mice aged 4-6 weeks at 1 × 10^6^ cells/mouse via subcutaneous injection on the right flank. After about 3 weeks, the tumor size was approximately 1 cm. Mice were randomized and divided in 3 different groups: no treatment, CLCN1 D275, and CLCN2 D275. Green fluorescence CLCNs were administered intravenously at 10 mg/kg via tail vein injection. After 24 hours, the mice were euthanized, and tumor, and major organs (liver, spleen, brain, lung, and kidney) were collected. The fluorescent signal of CLCNs in organs and tumor was detected by fluorescence microscopy and flow cytometry analysis. Briefly, for fluorescence microscopy studies, the whole tissue was embedded in OCT medium and frozen in dry ice; 5- to 15-μm-thick sections were cut at −20°C and transferred to a microscope slide at room temperature. The slides were imaged with use of a fluorescence microscope (LEICA DM5500 B) equipped with a FITC filter to visualize the green fluorescence of CLCNs. For flow cytometry analysis, organs and tissues were mechanically disaggregated by using 70-μm and 35-μm cell strainers to generate a single-cell suspension in PBS and analyzed by flow cytometry as described above.

### *In vivo* CLCNs-miR30b biodistribution

The 4- to 6-week-old nu/nu female mice bearing H1299 subcutaneous tumors were treated with CLCNs-miR30b and CLCNs-negative siRNA control at 1.5 mg/kg via tail vein injection. After 24 hours, the mice were euthanized, and tumors and major organs (liver, spleen, brain, lung, and kidney) were collected and stored in RNAlater solutions for RNA stabilization at -80°C. Total RNA was extracted from tissue samples by using TRIzol® RNA Isolation Reagents (Thermo Fisher Scientific). Reverse transcription was performed by using a High-Capacity cDNA Reverse Transcription Kit (Applied Biosystems) and miR30b RT primers. Quantitative real-time PCR (qPCR) was performed by using TaqMan MicroRNA Assays (Life Technologies) on a CFX384 Real-Time System (Bio-Rad) and miR30b TM primers.

### Chemical and histological analysis of blood and major organs

Nu/nu female mice bearing subcutaneous tumors on the right flank, were randomized and divided into 3 different groups: no treatment, CLCN1 D275, and CLCN2 D275. Green fluorescence CLCNs were administered intravenously at 10 mg/kg via tail vein injection. After 24 hours, the mice were euthanized, and blood, tumor, and major organs (liver, spleen, brain, lung, and kidney) were collected. Blood was tested for liver or kidney function alteration by routine chemical analysis. The whole tissue was embedded in OCT medium and frozen in dry ice; 5- to 15-μm-thick sections were cut at −20°C and transferred to a microscope slide at room temperature. Sections were stained for hematoxylin and eosin (H & E). The tissue sections were evaluated by a pathologist (AP) without knowledge of the treatment groups.

### *In vivo* CLCN2-miR150 inhibitor systemic administration

H1299 cells were injected into the flank of 6-8-week old nu/nu mice. After 2 weeks all mice developed a subcutaneous tumor and were randomized and divided in 2 groups (n= 5 mice each group). One group was treated for one week with 3 intravenous injections by tail vein of CLCN2-miR150 inhibitor at 1.5 mg/kg and one group was used as a control group. Tumors were measured 3 times a week for 3 weeks, and the growth rate was compared among the two treatment groups using generalized linear regression models to account for inter-mouse variability and the longitudinal nature of the data.

### Statistical analysis

All the numeric data are the result of a minimum of three independent experiments. Statistical computation was performed with Prism GraphPad software. The statistical significance was calculated with use of a two-tailed unpaired Student *t* test. SAS version 9.4 and S-Plus version 8.04 are used to carry out the computations for *in vivo* data analyses.

## Abbreviations

RNAi: RNA interference
siRNAs: small interfering RNAs
miRNAs: microRNAs
CLCN: cationic liquid crystalline nanoparticle
DOTAP: 1, 2-dioleoyl-3-trimethylammonium-propane (chloride salt)
GMO: glyceryl monooleate
TEM: transmission electron microscope
DLS: dynamic light scattering
NTA: nanoparticle tracking analysis
PDI: polydispersity index
GFP: green fluorescent protein
DAPI: 4', 6-diamidino-2-phenylindole, dihydrochloride
ER: endoplasmic reticulum
OCT: optimal cutting temperature.
